# Analysis and functional annotation of expressed sequence tags (ESTs) from multiple tissues of oil palm (*Elaeis guineensis *Jacq.)

**DOI:** 10.1186/1471-2164-8-381

**Published:** 2007-10-22

**Authors:** Chai-Ling Ho, Yen-Yen Kwan, Mei-Chooi Choi, Sue-Sean Tee, Wai-Har Ng, Kok-Ang Lim, Yang-Ping Lee, Siew-Eng Ooi, Weng-Wah Lee, Jin-Ming Tee, Siang-Hee Tan, Harikrishna Kulaveerasingam, Sharifah Shahrul Rabiah Syed Alwee, Meilina Ong Abdullah

**Affiliations:** 1 Department of Cell and Molecular Biology, Faculty of Biotechnology and Biomolecular Sciences, Universiti Putra Malaysia, 43400 UPM-Serdang, Selangor, Malaysia; 2 Advanced Biotechnology and Breeding Centre, Malaysian Palm Oil Board (MPOB), 6 Persiaran Institusi, Bandar Baru Bangi, Selangor, Malaysia; 3 KOOPrime Technologies (M) Sdn Bhd, 729, Block B, Jalan PJS 8/5, Mentari Business Park, Dataran Mentari, 46150 Petaling Jaya, Selangor, Malaysia; 4 Sime Plantations Sdn. Bhd., Wisma CONSPLANT 1, 2, Jalan SS 16/4, 47500 Subang Jaya, Selangor, Malaysia; 5 Sime Darby Technology Centre Sdn. Bhd., 2, Jalan Tandang, 46050 Petaling Jaya, Selangor, Malaysia; 6 Felda Agricultural Services Sdn Bhd., 7th Floor, Balai Felda, Jalan Gurney 1, 54000 Kuala Lumpur, Malaysia; 7 Interscience Sdn Bhd. 26 & 28, Jalan 25/34 Taman Mayang, 47301 Petaling Jaya, Selangor, Malaysia; 8 Hubrecht Institute, Uppsalalaan 8, 3584 CT Utrecht, The Netherlands; 9 Institut fur Genetics, University zu Koln, Zulpicherstr. 47, 50674 Koln, Germany; 10 Friedrich Miescher Institute, Maulbeerstrasse 66, CH 4058, Basel, Switzerland; 11 Laboratory of Applied Human Genetics, Division of Medical Sciences, National Cancer Centre of Singapore, 11, Hospital Drive, 169610, Singapore

## Abstract

**Background:**

Oil palm is the second largest source of edible oil which contributes to approximately 20% of the world's production of oils and fats. In order to understand the molecular biology involved in *in vitro *propagation, flowering, efficient utilization of nitrogen sources and root diseases, we have initiated an expressed sequence tag (EST) analysis on oil palm.

**Results:**

In this study, six cDNA libraries from oil palm zygotic embryos, suspension cells, shoot apical meristems, young flowers, mature flowers and roots, were constructed. We have generated a total of 14537 expressed sequence tags (ESTs) from these libraries, from which 6464 tentative unique contigs (TUCs) and 2129 singletons were obtained. Approximately 6008 of these tentative unique genes (TUGs) have significant matches to the non-redundant protein database, from which 2361 were assigned to one or more Gene Ontology categories. Predominant transcripts and differentially expressed genes were identified in multiple oil palm tissues. Homologues of genes involved in many aspects of flower development were also identified among the EST collection, such as *CONSTANS*-*like*, *AGAMOUS-like (AGL)2, AGL20, LFY-like, SQUAMOSA, SQUAMOSA binding protein (SBP) *etc. Majority of them are the first representatives in oil palm, providing opportunities to explore the cause of epigenetic homeotic flowering abnormality in oil palm, given the importance of flowering in fruit production. The transcript levels of two flowering-related genes, *EgSBP *and *EgSEP *were analysed in the flower tissues of various developmental stages. Gene homologues for enzymes involved in oil biosynthesis, utilization of nitrogen sources, and scavenging of oxygen radicals, were also uncovered among the oil palm ESTs.

**Conclusion:**

The EST sequences generated will allow comparative genomic studies between oil palm and other monocotyledonous and dicotyledonous plants, development of gene-targeted markers for the reference genetic map, design and fabrication of DNA array for future studies of oil palm. The outcomes of such studies will contribute to oil palm improvements through the establishment of breeding program using marker-assisted selection, development of diagnostic assays using gene targeted markers, and discovery of candidate genes related to important agronomic traits of oil palm.

## Background

The oil palm (*Elaeis guineensis *Jacq.) is a perennial monocotyledonous plant which belongs to the family Arecaceae originating from West Africa. The fruit pulp and nut that provide palm and kernel oil, respectively; made oil palm a high yielding oil-producing crop [1]. At present, palm oil production is second only to that of soybean oil in terms of world vegetable oil production and the demand for palm oil is expected to increase in future. In order to meet the increasing demand for palm oil, an improvement in yield is required.

Clonal propagation of oil palm via tissue culture has been developed for mass propagation of elite planting materials. Although this approach has been widely used in the oil palm industries, the embryogenesis rate is low and a proportion of the tissue culture derived plants exhibited abnormalities. Therefore, it is important to understand the molecular events that happened during somatic embryogenesis and *in vitro *culture to improve the production scale and cost efficiency of the tissue culture process. In addition, the occurrence of abnormal fruit type known as *mantled *[2] has reduced the number of fertile fruits in palms propagated by tissue culture, thus resulting in loss of oil yield.

Root plays an important role in water and nutrient uptake from the soil. It also serves as an anchorage for plant and secretes root exudates with growth regulatory properties into the rhizosphere. Oil palm roots are usually infected by mycorrhizal fungus that assist the uptake of nutrients especially phosphate. Basal stem rot (BSR) caused by *Ganoderma boniensis *is a major disease in oil palm roots. The fungus attack the root of oil palm causing trunk rot. This disease remains to be the major constrain to sustainable palm oil production, causing significant yield losses either by direct loss of diseased palms or reduced yield of infected palms, in addition to requirement for earlier replanting [3]. Approximately 30–70% of oil palms are lost due to BSR by the end of each planting cycle, and the damage occurs increasingly early from one planting cycle to the next [4]. Understanding root physiology, diseases and symbiotic relationships will contribute towards the economical growth of healthy palms.

Single-pass sequencing of the 5' and/or 3' ends of randomly selected cDNA clones, is an effective approach to provide genetic information of an organism. These sequences can serve as markers or tags for transcripts, and have been used in the development of markers for reference genetic map and recovery of full-length cDNA and genomic sequences. Expressed sequence tags (ESTs) are also useful for the discovery of novel genes, investigation of genes of unknown function, comparative genomic study, and recognition of exon/intron boundaries. Currently, there are less than 3000 available oil palm sequences in the GenBank, and majority of these sequences are ESTs which had been reported by Jouannic *et al*. [5]. The lack of sequence information has limited the progress of gene discovery and characterisation, global transcript profiling, probe design for development of gene arrays, and generation of molecular markers for oil palm.

In this study, we have generated and analysed more than 14000 ESTs from oil palm zygotic embryos, suspension cells, shoot apical meristems, young and mature flowers, and roots. The availability of these EST sequences will allow comparative genomic studies between oil palm and other monocotyledonous and dicotyledonous plants, development of molecular markers for the establishment of reference genetic map, design and construction of cDNA microarray for global gene expression profiling.

## Results

A total of six cDNA libraries were constructed from multiple oil palm tissues including zygotic embryos, suspension cell cultures, shoot apical meristems, young and mature flowers and roots; for the generation of ESTs from oil palm. The primary titer of all cDNA libraries used in this study consisted of at least 10^6 ^clones with more than 90% recombinant clones as revealed by X-Gal/IPTG screening (Table 1).

**Table 1:** Summary of the oil palm cDNA libraries used in this study

**Library**	**Source of tissues**	**Titer of cDNA library (pfu/ml)**	**Percentage of recombinant clones (%)**
Root	Root tissues of 3 month-old seedlings	5.08 × 10^9^	92.87
Shoot apical meristem	Shoot apical meristem tissues of 6 month-old seedlings	5.43 × 10^9^	92.25
Young flower	Male and female flowers of 4–6 cm	6.4 × 10^9^	90
Mature flower	Female flowers of 26 cm	3.19 × 10^9^	97
Suspension cell culture	Suspension cell culture	1.3 × 10^12^	98
Zygotic embryo	Zygotic embryo	9.25 × 10^10^	90

In this study, two approaches were employed in the selection of cDNA clones for the generation of ESTs. In the first approach, cDNA clones were isolated randomly from each cDNA library after mass excision whereas 'cold' plaque screenings were performed in the second approach. In the latter approach, the cDNA library of suspension cell culture was hybridized with the first strand cDNA of suspension cell culture; whereas the young flower, mature flower and shoot apical meristem libraries were hybridized with the cDNA of young leaves and young or mature flowers or shoot apical meristems, respectively. Only cDNA clones that did not show positive signals in the respective hybridizations were selected for sequencing. This approach was aimed to increase the possibility of isolating rare sequences in respective cDNA libraries.

In total, 14537 ESTs from single-pass 5' sequencing of 16149 cDNA clones (GenBank: EL680967 – EL695503) passed the quality control for high confidence base call with an average read length of approximately 600 bp. Among these ESTs, 3772 were generated from 'cold' plaque screening whereas the remaining ESTs were generated from cDNA clones that were isolated randomly. The GC content of the EST sequences was approximately 48%. Approximately 56% of the sequences appeared twice or more times among the ESTs. The EST sequences comprised an estimated 2129 tentative unique contigs (TUCs, see additional file 1) and 6464 tentative unique sequences (TUSs) (Table 2).

**Table 2:** Summary of ESTs from oil palm

	**Number**	**%**
Total ESTs sequenced	16149	-
Number of EST sequences with readable sequence	14537^a^	-
Redundant sequences	8073	-
Number of tentatively unique genes (TUGs)	8593	-
Number of unique sequences (TUSs, singletons)	6464	75.2^b^
Number of tentatively unique contigs (TUCs)	2129	24.8^b^
TUGs with significant matches	6008	69.9^b^
TUGs with non-significant matches	2585	30.1^b^

^a^This number consists of 4943 ESTs from root, 1854 from shoot apical meristem, 2623 from mature flower, 2807 from young flower, 1723 from suspension cell culture and 588 from zygotic embryo,

^b^Percentage of TUGs.

Among these sequences, approximately 70% had significant matches with sequences in the non-redundant protein database based on an E value cut off which was equal or less than 10^-5^, 20% with non-significant matches and 10% had no matches to sequences in the non-redundant protein database in GenBank. The percentages of oil palm sequences with significant matches varied from 63% in the root tissues to 78% in the oil palm suspension cell culture. Comparing the oil palm TUGs against the EST database using BLASTN demonstrated that the percentage of oil palm sequences that had significant matches was 39% based on an E value cut off which was equal or less than 10^-5^. Less than 4% of oil palm ESTs had no matches to the sequences in the EST database. These sequences may represent the novel sequences in oil palm.

The number of ESTs in TUCs ranged from 2 to 145 with more than 54% TUCs consisting of 2 ESTs, 18% with 3 ESTs and 25% with 4 – 12 ESTs. The top 20 most highly expressed genes (Table 3) accounted for 6 % of the sequence reads. The most widely expressed genes encoded for chaperonin 60 which was present in five cDNA libraries, whereas cyclophilin and glyceraldehyde 3-phosphate dehydrogenase were present in all six cDNA libraries. Among the predominant transcripts with more than 25 ESTs were glycine-rich RNA binding protein, alpha-tubulins, metallothionine-like proteins, PVR3-like protein, DNA J-like protein which were previously reported as transcripts that predominated the oil palm ESTs by Jouannic *et al*. [5]. However, other highly expressed proteins such as putative translation initiation factor and elongation factor were also identified and majority of them were involved in the housekeeping functions of cell. One of the highly expressed genes had no significant homology to the public database.

**Table 3:** List of transcripts that predominate the oil palm TUCs

						**Number of EST in each cDNA library**					
**Contig ID**	**Putative identity**	**No of ESTs**	**Species**	**Accession number**	**E-value**	**Root**	**Shoot apical meristem**	**Young flower**	**Mature flower**	**Suspension cell culture**	**Zygotic embryo**
Contig1765	Metallothionein-like protein	145	*Typha latifolia*	gb|AAK28022.1	1E-60	50	8	35	39	13	0
Contig962	Glycine-rich RNA binding protein 2	59	*Pelargonium × hortorum*	gb|AAB63581.1	8E-36	6	8	22	20	1	2
Contig1185	Putative translation initiation factor eIF-1A-like	57	*Solanum tuberosum*	gb|ABB55392.1	8E-51	21	3	16	17	0	0
Contig1589	Alpha-tubulin	49	*Prunus dulcis*	emb|CAA47635.1	0.0	5	6	9	28	1	0
Contig447	Cyclophilin	43	*Ricinus communis*	emb|CAC80550.1	6E-77	16	5	11	7	2	2
Contig1818	Type 2 metallothionein-like protein	43	*Typha angustifolia*	gb|ABQ14530.1	2E-10	41	0	0	0	2	0
Contig1809	Putative translation elongation factor eEF-1 beta' chain	42	*Oryza sativa*	dbj|BAC22427.2	3E-63	7	1	10	24	0	0
Contig1384	Translationally controlled tumor protein	42	*Elaeis guineensis*	gb|AAQ87663.1	7E-88	9	7	14	9	0	3
Contig165	Putative OsCTTP	41	*Oryza sativa*	dbj|BAD19560.1	5E-84	17	0	21	3	0	0
Contig1595	Type 2 metallothionein-like protein	41	*Typha latifolia*	gb|AAK28022.1	8E-11	2	6	12	21	0	0
Contig2050	hypothetical protein OsI_022576	37	*Oryza sativa*	gb|EAZ01344.1	4E-45	17	1	4	15	0	0
Contig1214	PVR3-like protein	36	*Ananas comosus*	gb|AAM28295.1	2E-17	14	9	3	10	0	0
Contig268	DnaJ-like protein	35	*Oryza sativa*	dbj|BAD25681.1	3E-177	4	14	9	5	3	0
Contig614	Early-methionine-labelled polypeptide	32	*Elaeis guineensis*	gb|ABD66069.1	1E-43	0	0	0	0	0	32
Contig2086	Cationic peroxidase 2	30	*Glycine max*	gb|AAC83463.1	1.E-155	3	0	5	10	12	0
Contig80	Glyceraldehyde 3-phosphate dehydrogenase	29	*Magnolia quinquepeta*	emb|CAA42905.1	2E-1744	2	5	4	4	12	2
Contig1135	Hypothetical protein CBG17156	29	*Caenorhabditis briggsae*	emb|CAE70527.1	0.22	9	14	0	0	5	1
Contig449	T6D22.2	27	*Arabidopsis thaliana*	gb|AAF79822.1	0.0	2	9	10	6	0	0
Contig584	Myo-inositol-1-phosphate synthase	26	*Nicotiana tabacum*	dbj|BAA95788.1	0.0	0	3	12	11	0	0
Contig1454	Alpha-tubulin	26	*Prunus dulcis*	emb|CAA47635.1	0.0	7	8	4	6	1	0

The most highly expressed genes in each cDNA library were listed in Table 4. The number of unique TUGs in each oil palm cDNA library that did not overlap with TUGs from other oil palm cDNA libraries were 2654, 940, 1193, 1094, 1027 and 299 for root, apical shoot meristem, young flower, mature flower, suspension cell culture and zygotic embryo, respectively. However, there were only 27 differentially expressed TUCs in multiple cDNA libraries according to the R statistic [6] with Bonferroni correction at the significance threshold of 2.35 × 10^-5 ^(Table 5). Among these are a few TUCs that were predominant in individual cDNA libraries such as acidic class III chitinase, thaumatin-like protein 1 and 1-aminocyclopropane-1-carboxylic acid oxidase in the suspension cell culture; early methionine-labelled polypeptides, 7S globulin, dehydrin-like protein, embryogenesis abundant protein D-34 and chaperone in the zygotic embryo; and putative flavonol 3-sulfotransferase STF-1 in the young flowers. In addition, the results also demonstrated higher copy number of certain transcripts in both young and mature flower such as myo-inositol 1-phosphate synthase, alpha tubulin, translation elongation factor eEF-1, glycine-rich RNA binding protein and polyphenol oxidase. Both root and flower tissues (young and mature flowers) had high copy number of transcripts encoding for type 2 metallothionein-like proteins, however, the nucleotide sequences of these two contigs were different.

**Table 4:** List of transcripts that predominate in different oil palm tissues

**Putative identity**	**No of ESTs**	**Species**	**Accession number**	**E-value**
**Root**				
Type 2 metallothionein-like protein	93	*Typha latifolia*	gb|AAL09705.1	9E-10
Sucrose synthase	21	*Oncidium cv. 'Goldiana'*	gb|AAM95943.1	0.0
Putative translation initiation factor eIF-1A-like	21	*Solanum tuberosum*	gb|ABB55392.1	4E-51
Putative OsCTTP	17	*Oryza sativa*	ref|XP_465531.1	3E-84
OSJNBa0006M15.20	17	*Oryza sativa*	ref|XP_472724.1	2E-45
Cyclophilin	16	*Ricinus communis*	emb|CAC80550.1	2E-77
Cytochrome b5 domain-containing protein-like	15	*Oryza sativa*	ref|XP_468235.1	4E-16
PVR3-like protein	14	*Ananas comosus*	gb|AAM28295.1	6E-18
Putative ubiquitin-conjugating enzyme	11	*Oryza sativa*	dbj|BAD34325.1	3E-79
				
**Shoot apical meristem**				
rRNA intron-encoded homing endonuclease	16	*Pan troglodytes*	ref|XP_525925.1|	8E-07
DnaJ-like protein	15	*Oryza sativa*	emb|CAC39071.1	E-178
Hypothetical protein CBG17156	14	*Caenorhabditis briggsae*	emb|CAE70527.1	0.13
Putative fiber protein Fb2	10	*Oryza sativa*	ref|XP_465147.1	1E-56
Ethylene response factor	9	*Manihot esculenta*	gb|AAX84670.1	9E-73
Hypothetical protein FG07171.1	9	*Gibberella zeae PH-1*	gb|EAA76630.1	6.8
Type 2 metallothionein-like protein	9	*Typha latifolia*	gb|AAL09705.1	2E-20
Annexin p33	9	*Zea mays*	emb|CAA66900.2	E-116
Alpha-tubulin	9	*Prunus dulcis*	emb|CAA47635.1	E-124
Glycine-rich RNA binding protein 2	8	*Pelargonium × hortorum*	gb|AAB63582.1	4E-36
PVR3-like protein	8	*Ananas comosus*	gb|AAM28295.1	8E-18
Drought-induced protein like	8	*Arabidopsis thaliana*	emb|CAB10370.1	4E-10
				
**Young flowers**				
Cytoplasmic ribosomal protein S15a	45	*Daucus carota*	gb|AAK30203.1	4E-67
Glycine-rich RNA binding protein 2	22	*Pelargonium × hortorum*	gb|AAB63582.1	5E-36
Putative OsCTTP	21	*Oryza sativa*	ref|XP_465531.1	3E-84
Putative translation initiation factor eIF-1A-like	17	*Solanum tuberosum*	gb|ABB55392.1	4E-51
Translationally controlled tumor protein	14	*Elaeis guineensis*	gb|AAQ87663.1	3E-88
Putative STF-1	12	*Oryza sativa*	dbj|BAD31135.1	9E-67
Type 2 metallothionein-like protein	12	*Typha latifolia*	gb|AAL09705.1	6E-11
Ribosomal protein L15	11	*Oryza sativa*	ref|NP_909841.1	8E-92
Polyphenol oxidase	11	*Vitis vinifera*	emb|CAA81798.1	E-105
Cyclophilin	11	*Ricinus communis*	emb|CAC80550.1	2E-77
				
**Mature flower**				
Type 2 metallothionein-like protein	60	*Typha latifolia*	gb|AAL09705.1	8E-11
Alpha-tubulin	30	*Prunus dulcis*	emb|CAA47635.1	0.0
Putative translation elongation factor eEF-1 beta' chain	24	*Oryza sativa*	ref|NP_910927.2	5E-64
Glycine-rich RNA binding protein 2	20	*Pelargonium × hortorum*	gb|AAB63582.1	4E-36
Putative translation initiation factor eIF-1A-like	18	*Solanum tuberosum*	gb|ABB55392.1	4E-51
Similar to mucin 17	17	*Rattus norvegicus*	ref|XP_578244.1	0.004
OSJNBa0006M15.20	15	*Oryza sativa*	ref|XP_472724.1	2E-45
Cationic peroxidase 2	10	*Glycine max*	gb|AAC83463.1	E-155
				
**Suspension cell culture**				
Lipid transfer protein homolog	21	*Triticum aestivum*	gb|AAB32995.1	3E-25
Cationic peroxidase 2	12	*Glycine max*	gb|AAC83463.1	E-153
Glyceraldehyde 3-phosphate dehydrogenase	12	*Magnolia quinquepeta*	emb|CAA42905.1	E-174
Hypothetical protein	10	*Oryza sativa*	dbj|BAD87021.1	7E-27
Ubiquitin	10	*Antirrhinum majus*	emb|CAA48140.1	E-121
Non-symbiotic hemoglobin class 1	9	*Malus × domestica*	gb|AAP57676.1	2E-67
Putative beta-expansin	9	*Triticum aestivum*	dbj|BAD06319.1	2E-72
TAPETUM DETERMINANT 1	9	*Arabidopsis thaliana*	ref|NP_974612.1	4E-32
1-Aminocyclopropane-1-carboxylic acid oxidase	9	*Elaeis guineensis*	gb|AAP13098.1	E-168
Thaumatin-like protein precursor	7	*Malus × domestica*	gb|AAC36740.1	2E-82
				
**Zygotic embryo**				
Early-methionine-labelled polypeptide	46	*Secale cereale*	emb|CAB88095.1	2E-35
7S globulin	21	*Elaeis guineensis*	gb|AAK28402.1	0.0
Dehydrin-like protein	10	*Elaeis guineensis*	gb|AAF60172.1	1E-26
Late embryogenesis abundant protein D-34	9	*Gossypium hirsutum*	sp|P09444	4E-76
Protein, small heat shock	6	*Codonopsis lanceolata*	gb|AAW02791.1	1E-23
Seed maturation protein LEA 4	4	*Glycine tomentella*	gb|AAG37452.1	6E-24
Translationally controlled tumor protein	4	*Elaeis guineensis*	gb|AAQ87663.1	3E-88
Ribosomal protein S27-like protein	4	*Solanum tuberosum*	gb|ABA40465.1	8E-35
Class II metallothionein	4	*Zea mays*	emb|CAA84233.1	5E-17

**Table 5:** Differentially expressed genes with the top hits (R values) in multiple cDNA libraries of oil palm

				**Number of EST in each cDNA library**						
**Contig ID**	**Description**	**E-value**	**Organism**	**Root**	**Shoot apical Meristem**	**Young Flower**	**Mature Flower**	**Suspension cell culture**	**Zygotic embryo**	**R value**
Contig165	Putative OsCTTP	2.93E-84	ref|XP_507481.1*Oryza sativa*	17	0	21	3	0	0	0
Contig1512	Hypothetical protein XP_524016	1.00E-170	ref|XP_524016*Pan troglodytes*	0	0	0	20	1	0	0
Contig584	Myo-inositol-1-phosphate synthase	1.00E-170	dbj|BAA95788.1*Nicotiana tabacum*	0	3	12	11	0	0	0
Contig1589	Alpha-tubulin	1.00E-11	emb|CAA47635.1*Prunus dulcis*	5	6	9	28	1	0	0
Contig1595	Type 2 metallothionein-like protein	1.00E-07	gb|ABQ14530.1*Typha angustifolia*	2	6	12	21	0	0	0
Contig504	Acidic class III chitinase OsChib3a	1.00E-94	NP_917360.1*Oryza sativa*	0	0	0	0	13	0	0
Contig874	1-Aminocyclopropane-1-carboxylic acid oxidase	1.00E-168	gb|AAP13098.1*Elaeis guineensis*	0	0	0	0	9	0	0
Contig1135	Hypothetical protein	0.130103	emb|CAE70527.1*Caenorhabditis briggsae*	9	14	0	0	5	1	0
Contig1435	rRNA intron-encoded homing endonuclease	1.00E-08	ref|XP_525925.1*Pan troglodytes*	0	14	1	1	0	0	0
Contig1809	Translation elongation factor eEF-1 beta' chain	1.00E-65	ref|XP_506540.1*Oryza sativa*	7	1	10	24	0	0	0
Contig1818	Type 2 metallothionein-like protein	1.00E-10	gb|AAL09705.1*Typha latifolia*	41	0	0	0	2	0	0
Contig614	Early-methionine-labelled polypeptide	1.00E-170	emb|CAB88095.1*Secale cereale*	0	0	0	0	0	32	0
Contig611	Early-methionine-labelled polypeptide	1.00E-36	emb|CAB88095.1*Secale cereale*	0	0	0	0	0	12	0
Contig634	7S globulin	1.00E-25	gb|AAK28402.1*Elaeis guineensis*	0	0	0	0	0	21	0
Contig1007	Dehydrin-like protein	1.00E-26	gb|AAF60172.1*Elaeis guineensis*	0	0	0	0	2	10	0
Contig1024	Embryogenesis abundant protein D-34 (LEA D-34)	1.00E-76	sp|P09444*Gossypium hirsutum*	0	0	0	0	0	9	0
Contig1097	Chaperone	5E-26	gb|ABF61872.1*Agave tequilana*	0	0	0	0	0	6	0
Contig530	Polyubiquitin	3E-121	gb|ABU40645.1*Triticum aestivum*	2	0	0	0	10	0	0.000001
Contig962	Glycine-rich RNA binding protein 2	1.00E-36	gb|AAB63582.1*Pelargoniu × hortorum*	6	8	22	20	1	2	0.000001
Contig775	Polyphenol oxidase	1.00E-105	emb|CAA81798.1*Vitis vinifera*	0	0	12	3	0	0	0.000002
Contig701	Unknown protein	1.00E-38	gb|ABF59206.1*Arabidopsis thaliana*	0	0	2	0	9	0	0.000002
Contig2113	Putative STF-1	1.00E-72	dbj|BAD31135.1*Oryza sativa*	1	0	12	0	0	0	0.000002
Contig2086	Cationic peroxidase 2	1.00E-156	gb|AAC83463.1*Glycine max*	3	0	5	10	12	0	0.000004
Contig546	Non-symbiotic hemoglobin class 1	1.00E-67	gb|AAP57676.1*Malus × domestica*	3	0	0	0	9	0	0.000008
Contig553	OSJNBa0010H02.8	1.00E-73	gb|AAK84683.1*Oryza sativa*	1	0	0	0	8	0	0.000015
Contig659	Thaumatin-like protein 1 – apple tree	1.00E-82	gb|AAC36740.1*Malus × domestica*	0	0	0	0	7	0	0.000016

More detailed functional annotation was performed by mapping tentative unique genes (TUGs) to the Gene Ontology Consortium structure which provides a structured and controlled vocabulary to describe gene products according to three ontologies: cellular components, biological processes and molecular functions. The GO classifications of TUGs from oil palm were summarized in Table 6, according to their involvement in various biological processes, molecular functions and cellular localization. In total, 2361 TUGs could be mapped to one or more ontologies, 2376 assignments were made to the category of molecular function (level 3), with approximately 42% in binding (including nucleotide, ion, nucleic acid, protein, and cofactor binding); 41% in catalytic activities (including transferase, hydrolase and oxidoreductase activities); 7% in structural constituents of ribosome; and 5% in transport activities (ion and carrier transport). Under the category of biological process, 4583 assignments (level 4) were made to cellular metabolism (23%), primary metabolism (21%), macromolecule metabolism (16%) and biosynthesis (9%). Finally, for cellular components, the vast majority of 1948 assignments (level 4) were assigned to the intracellular (40%) and intracellular parts (38%). The GO assignments of TUGs from each cDNA library were also summarized in Table 6. In general, the main GO categories assigned to TUGs from individual cDNA libraries were similar to that of the overall analysis mentioned above. The minor differences in percentage may be non-significant as only some of the TUGs were shown to be differentially expressed in multiple tissues (Table 5).

**Table 6:** Gene Ontology (GO) classifications of TUGs from oil palm according to their involvement in biological processes, molecular functions and cellular localizations

	**Percentage in each cDNA library (%)**						
**Gene ontology (GO) classification**	**Root**	**Shoot apical meristem**	**Young flower**	**Mature flower**	**Suspension cell culture**	**Zygotic embryo**	**Overall**
**Biological processes**							
Biosynthesis	9	10	9	9	7	13	9
Catabolism	2	1	2	2	4	3	2
Cell organization and biogenesis	3	3	4	4	3	3	3
Cellular metabolism	23	23	23	23	22	22	23
Establishment of localization	7	6	5	5	7	5	7
Macrmolecule metabolism	16	17	18	19	17	19	16
Nitrogen compound metabolism	2	2	1	1	2	1	2
Primary metabolism	21	22	22	22	21	22	21
Protein localization	1	1	1	1	1	3	1
Regulation of cellular physiological process	2	2	2	2	2	1	2
Regulation of metabolism	2	2	2	2	2	< 1	2
Response to chemical stimulus	1	2	1	1	1	1	1
Transport	7	6	5	5	6	5	7
Others	4	3	5	4	5	2	4
							
**Molecular function**							
Antioxidant activity	1	1	1	2	3	1	1
Binding							
Cofactor binding	2	2	1	2	2	3	2
Ion binding	3	9	11	12	12	13	11
Nucleic acid binding	9	15	15	13	11	12	11
Nucleotide binding	11	15	15	12	9	11	13
Protein binding	6	6	8	5	6	4	5
Catalytic activity							
Hydrolase activity	11	8	10	10	10	8	11
Isomerase activity	2	2	2	2	2	2	2
Ligase activity	3	2	3	2	2	1	2
Lyase activity	3	2	1	2	3	3	3
Oxidoreductase activity	9	7	5	7	11	5	9
Peroxidase activity	1	1	1	1	2	0	1
Transferase activity	10	10	10	9	11	11	13
Structural constituent of ribosome	9	9	9	11	6	14	7
Translation regulator activity	1	3	3	2	1	4	2
Transcription regulator activity	1	2	1	1	2	0	1
Transport							
Carrier activity	2	2	1	1	2	1	2
Ion transport	3	2	1	1	2	1	3
Others	13	2	2	5	3	7	1
							
**Cellular component**							
External encapsulating structure	< 1	1	1	1	1	0	1
Intracellular	39	43	42	43	40	39	40
Intracellular part	37	41	40	42	39	38	38
Membrane	12	8	9	7	11	11	11
Membrane bound vesicle	1	1	1	1	2	1	1
Membrane part	7	6	5	5	6	6	7
Organelle inner membrane	1	< 1	1	< 1	< 1	1	1
Others	3	< 1	1	1	1	4	1

Table 7 provides a list of TUGs from oil palm that are homologous to genes known to be involved in flower development. The ESTs related to oil palm flowering included CONSTANS-like protein, GIGANTEA-like protein, AGL20-like MADS box transcriptional factor, LFY-like protein, SQUAMOSA protein, SQUAMOSA promoter binding protein, AP domain containing proteins and aintegumenta-like protein. These proteins have been reported to be involved in flowering time, determination of floral identities and development of floral organs. Majority of these proteins were the first representatives of their gene families from oil palm. The results demonstrated that EST approach was successful in uncovering homologues of many (90 or more) putative floral regulatory genes, supporting the hypothesis that this regulatory pathway is largely conserved in angiosperms. Some of these gene families were not exclusively expressed in young and mature flowers, but were also expressed in other tissues. For example, AP2 domain containing protein was expressed in zygotic embryo and root tissues as well. However, 5 and 11 MADS box proteins were found in young and mature flowers respectively; 7 shoot-meristemless protein, 5 ZF-HD homeobox and 3 AGL20 in young flowers; and 10 knotted -like homeobox protein in both floral libraries. Functional elucidation of these proteins may shed light on the flowering process of oil palm. Two of these cDNA candidates, EgSBP (GenBank: EL682671) and EgSEP (GenBank: EL686357) isolated from young and mature flowers respectively; were further characterised by Northern analysis.

**Table 7:** List of transcripts from oil palm that are homologous to genes involved in flower development and formation

		**Number of ESTs in**					
**Gene**	**Total EST**	**Root**	**Shoot apical meristem**	**Mature flower**	**Young flower**	**Suspension cell culture**	**Zygotic embryo**
AGL2	8	0	0	8	0	0	0
AGL20	3	0	0	0	3	0	0
Putative argonaute protein	4	0	2	0	0	2	0
AP2 domain containing protein	5	2	0	1	0	0	2
CONSTANS-like protein 1	1	0	0	0	1	0	0
Early flowering protein 1	3	1	0	1	0	1	0
Floral organ regulator 2	1	0	0	0	0	1	0
Flowering promoting factor-like 1	1	0	0	1	0	0	0
Putative gigantea	3	3	0	0	0	0	0
Knotted1-like homeobox protein	11	0	1	3	7	0	0
LFY-like protein	1	0	0	0	1	0	0
Myb-like protein	15	3	1	1	6	3	1
NAC family protein	11	2	3	0	0	6	0
NAM-like protein	2	1	0	0	1	0	0
Putative GAMYB-binding protein	4	1	3	0	0	0	0
Putative LHY protein	1	0	0	0	1	0	0
Scarecrow-like protein	5	1	2	0	0	2	0
Shoot meristemless-like protein	8	0	0	0	7	1	0
SQUAMOSA protein	7	2	0	3	2	0	0
SQUAMOSA binding protein	7	1	0	2	4	0	0
STYLOSA protein	2	1	0	0	0	1	0
YABBY-like transcription factor	5	3	1	1	0	0	0
ZF-HD homeobox protein	7	1	1	0	5	0	0

**Figure 1:**
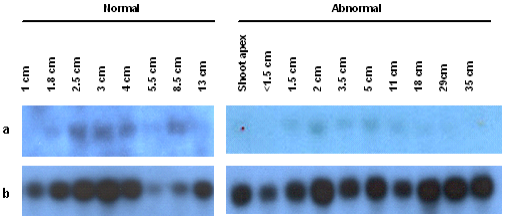
Transcriptional expression of *EgSBP *(a) in normal and abnormal male flowers of various sizes with cyclophilin (b) as a loading reference.

**Figure 2:**
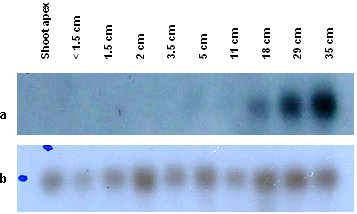
Transcriptional expression of *EgSEP *(a) in abnormal female flowers of various sizes with cyclophilin (b) as a loading reference.

In order to study the temporal expression of *EgSBP and EgSEP*, blots were prepared from RNA extracted from different stages of normal and abnormal flower representing early, mid and late period of flowering. The transcripts of *EgSBP *were detected in normal male flower of 1.8 cm and above. Figure 1 also shows low transcript levels of *EgSBP *in the abnormal flower of 1.5 cm and the signal intensities increased gradually during the subsequent developmental stages until the size of the flower reached 11 cm. On the other hand, *EgSEP *was expressed during late floral development and no expression was detected in the shoot apex (Figure 2). In the abnormal female flower, it was first detected at very low levels in flowers of 18 cm and the expression gradually increased as flower development progressed. The expression was at its highest in flowers of 35 cm (Figure 2). In the normal male flower, EgSEP was first detected in flowers of 19 cm (data not shown).

In addition to the flowering related genes, we also surveyed a few well-characterised biological processes and metabolic pathways to determine the extent to which such pathways were represented within the TUGs of oil palm. Important enzymes that are involved in the oil biosynthesis were represented by ESTs encoding for acetyl-CoA carboxylase, malonyl-CoA: ACP transacylase, beta-ketoacyl-ACP synthase, beta-ketoacyl-ACP dehydratase, enoyl ACP reductase, enoyl ACP hydratase, palmitoyl protein thioesterase and desaturases such as steroyl-ACP desaturase, omega 3 desaturase and omega 6 desaturase. Besides, there were also ESTs encoding for enzymes in the ascorbate-glutathione cycle including ascorbate peroxidase, monohydroascorbate reductase, dehydroascorbate reductase, glutathione reductase, glutathione S-transferase, glutathione peroxidase, Cu/Zn-superoxide dismutase and catalase; and enzymes involved in nitrogen utilization such as nitrate reductase, nitrate transporter, glutamine synthase, glutamate synthase and asparagine synthase.

## Discussion

In this study, we have analysed a complex data set of oil palm transcripts to gain insight into the gene content and to provide a preliminary assessment of the transcript and gene expression profile of this crop. The number of ESTs generated is more than 4.8 fold of the total number of entries for oil palm in GenBank. Despite the fact that more number of ESTs are required in order to have a good chance of finding any gene that is of interest, this may take probably several years before this number can be achieved. We have decided to report the existing number of ESTs as this sequence information is important to the science community since the oil palm genome has not been fully sequenced and the data is not currently available in the public databases.

Oil palm was represented by approximately 3000 sequences in the NCBI database prior to this study. Much of the previous EST sequencing in oil palm has focused on cDNAs derived from zygotic embryos, shoot apical meristems and flowers consistent with the overriding interest of the oil palm industry in yield improvement through clonal propagation of elite planting materials and reduction of losses attributed to abnormal epigenetic flowering. In this study, we have generated ESTs from oil palm tissues that were not covered by other research groups, most of the transcripts uncovered are the first representatives for oil palm especially those sequences from root and suspension cell culture. Eventhough the number of ESTs from flowers, shoot apical meristem and suspension cell culture was less, close to 50% of them were generated from 'cold' plaque screening. This approach increased the possibility of sequencing rare transcripts and tissue-specific ESTs. Overall, the GC content of the sequences is approximately 48 % which is close to the value reported by Jouannic *et al*. [5].

Since the oil palm materials used in this study were sampled from Dura × Pisifera hybrids developed by different companies and institution, there are possibilities that variations (including single nucleotide polymorphisms) may occur among the sequences encoding for the same transcript. Our preliminary screening of a few contigs assembled from high number of ESTs demonstrated some minor variations among the sequences. However, these sequences may have to be verified by resequencing to exclude any sequencing errors. In addition, we have also detected sequence variation in cDNAs encoding for type 2 metallothionein-like proteins in flower and root tissues, respectively. These EST sequences may serve as a source for tissue-specific markers or probes for the recovery of tissue-specific promoters.

In this study, we have surveyed several well-characterised biological processes and metabolic pathways to determine the extent to which such pathways were represented within the TUGs of oil palm. Our findings showed that the oil biosynthesis, ascorbate-glutathione cycle, nitrogen utilization and flowering pathways were well represented by ESTs encoding for some of the important proteins involved. We have also demonstrated that this EST data can be utilized not only for gene discovery but also for comparative analysis of gene expression within/between oil palm tissues. For example, methionine-labelled polypeptide (which also showed high identities to late embryogenesis abundant protein), 7S globulin, dehydrin-like protein and embryogenesis abundant protein D-34 that were predominant in zygotic embryos, were absent in suspension cell culture which was predominated by the transcripts of acidic class III chitinase and 1-aminocyclopropane-1-carboxylic acid oxidase. The differentially expressed genes in zygotic embryos indicated that they might be in a later developmental stage than the suspension cell culture although both tissues were embryogenic. These ESTs may have the potential to be developed as molecular markers for diagnostic assays, especially for tissue cultures. Besides, we also demonstrated that the transcripts encoding for polyphenol oxidase and flavonol 3-sulfotransferase were in high copy number in flowers. It is not surprising since both enzymes are involved in the flower coloration. In summary, our results showed that the differentially expressed transcripts reflected the physiological and developmental states of the oil palm tissues used for the construction of cDNA libraries. This information also provides a preliminary assessment of the gene expression profile of these genes in different oil palm tissues.

The oil palm homologue for *SEPALLATA, EgSEP*, was further characterized by northern analysis. *SEPALLATA *was shown to be required for petal, stamen and carpel identities thus necessary for the activities of the B and C function genes [7]. EgSEP is the most abundant transcription factor among the oil palm ESTs (7 clones) involved in flower development. It was expressed in normal male flowers with fully developed anthers and pollens (data not shown), and also in abnormal male flowers with fully developed supernumerary carpels [8]. However, EgSEP was not able to differentiate normal male flowers from abnormal male flowers with carpel-like structures. The results were consistent with the expression of oil palm *SEPALLATA *in both female and male flowers reported by Adam *et al*. [9].

EgSBP is a putative homologue for SQUAMOSA binding protein (SBP) which binds to the promoter of SQUAMOSA at unique sequence motif and involves in the control of early flower development [10]. *EgSBP *was first expressed in normal male oil palm flowers of 1.8 cm of which bracts have already developed around the rachillae. At this stage, the male rachillae can be differentiated from the female rachillae by the number and shape of the bracts. The expression of *EgSBP *increased in the subsequent flower developmental stages during the emergence of flower primordia (2 – 3 cm), emergence of male flowers in male rachillae (3 – 5 cm), and development of stamens (5 – 7 cm) and anther (7 – 9 cm). In abnormal flower, the development of supernumerary carpels occurs when the inflorescence is 5 – 7 cm [8]. Stamens of abnormal male flower develops into carpel-like structures at 7 cm and above. However, our results showed that the transcript profiles of *EgSBP *in normal and abnormal male flowers were similar (Figure 1). The onset of transcription of *EgSBP *is consistent with the findings of Cardon *et al*. [11] that some of the *SBP *genes were constitutively expressed during flower development. On the other hand, ESTs for SBP-like proteins were also identified in the root and mature flower tissues. It is not surprising as its target gene, *SQUAMOSA*, was also found to be expressed in vegetative tissue and did not have an expression pattern specific to either the male or female inflorescences [9].

Interestingly, the transcript for *GIGANTEA *– a circadian clock controlled gene that regulates photoperiodic flowering [12] was found in oil palm root instead of floral tissues. The role of this protein in root has not been characterised. On the other hand, the putative homologue for LHY which was proposed to function either within the circadian oscillator or in its output pathways, was found only in young flowers. In addition, ESTs for LFY-like, and CONSTANS-like, AGL20, SQUAMOSA proteins were also uncovered among the ESTs related to flowering. Among these sequences, only the cDNA sequence of SQUAMOSA have been isolated and examined in the vegetative and reproductive tissues of oil palm [9]. Further characterisation of these genes may enhance our understanding of the flowering pathway of oil palm which is largely unknown.

## Conclusion

The EST sequences generated in this study will be a source of gene-targeted and tissue-specific markers. The data will also facilitate the fabrication of DNA array for future studies of oil palm. Transcript profiling through microarray could further contribute towards the understanding of fundamental aspects in oil palm biology such as transcriptional responses correlated with embryogenesis, abnormal flowering, diseases etc. The outcomes of such studies will contribute to oil palm improvements through the establishment of breeding program using marker-assisted selection, development of diagnostic assays using gene-targeted markers, recovery of genomic sequences and discovery of candidate genes related to important agronomic traits of oil palm.

## Methods

### Plant materials

The oil palm materials (*Elaeis guineensis *Jacq.) used in this study were summarized in Table 1. Oil palm suspension cell cultures and shoot apical meristems from 6-month old oil palm seedlings were kindly provided by Applied Agricultural Research Sdn Bhd., Sungai Buloh, Malaysia; whereas the root tissues were obtained from 3-month old oil palm seedlings from Guthrie Sdn Bhd., Seremban, Malaysia. The young flowers (4–6 cm), mature flowers (26 cm) and zygotic embryos used in this study were provided by Malaysian Palm Oil Board, Bangi, Malaysia. All of the above materials were sampled from Dura × Pisifera hybrids developed by respective institution and companies.

### RNA extraction

RNA from oil palm shoot apical meristem tissues, young (male and female) and mature (female) flowers was extracted by using the method described by Rochester *et al*. [13], whereas RNA from oil palm suspension cells and zygotic embryos was extracted by using the method described by Schultz *et al*. [14]. CsCl gradient [15] was used to extract RNA from root tissues.

### cDNA library construction

Poly A^+ ^RNA isolation was performed using PolyA Tract Isolation System (Promega, USA) or μ MACS mRNA isolation kit (Miltenyi Biotec, Germany) according to the manufacturers' instructions. cDNA was prepared using a cDNA synthesis kit (Stratagene, USA) and directionally cloned into Uni-Zap vector (Stratagene, USA). The resulting primary cDNA library was amplified to more than 10^9 ^pfu/ml. Plasmids (pBluescript SK-) containing cDNA inserts were *in vivo *and mass-excised from phage stocks using ExAssist helper phage and propagated in *Escherichia coli *SOLR cells according to the manual provided by manufacturer.

### 'Cold' plaque screening

The cDNA library of suspension culture was screened with a ^32^P-labelled probe prepared from the first-strand cDNA of suspension culture. The young and mature female flower libraries were screened twice with ^32^P-labelled probes prepared from the cDNA of young leaves and young or mature flowers, respectively. The shoot apical meristem library was screened with cDNA from young leaves and shoot apical meristems, respectively. These probes were prepared from PCR amplified cDNA using SMART cDNA synthesis kit (Clontech, USA). Hybridization was performed in 5 × SSPE, 5 × Denhardt's solution, 0.5% (w/v) SDS, 100 μg/ml denatured herring sperm DNA at 60°C. Membranes were washed in 2 × SSC, 0.1% SDS at room temperature twice for 10 minutes each before the membranes were exposed to autoradiography films at -80°C. The 'cold' plaques that did not show any signals upon secondary screening were *in vivo *excised.

### Nucleotide sequencing

The cDNA clones were cultured for plasmid DNA preparation manually [16] or by using Perfectprep^® ^Plasmid 96 Vac kit (Eppendorf, Germany) according to the manual provided by the manufacturer. Automated cycle sequencing was performed by using T3 universal primer and BigDye Terminator (Applied Biosystems, USA) or ET Terminator (Amersham Pharmacia Bioscience, USA), and electrophoresed on DNA sequencer ABI 3730XL or ABI PRISM 377 (Applied Biosystems, USA) or Megabace™ 500 (Amersham Biosciences, USA).

### Clustering analysis and annotation

Quality control of raw DNA sequences was performed by using Phred program [17] to remove sub-standard reads, followed by Lucy2 [18] to eliminate the vector and adapter sequences. Contig Assembly Program 3 (CAP3) was used to cluster the overlapping ESTs into contigs [19]. The edited EST was translated into six reading frames and compared with the non-redundant protein database at the National Center for Biotechnology Information (NCBI) using the default setting of BLASTX program [20]. BLASTN program was used to compare the nucleotide sequences with the sequences in the EST database at NCBI. BLASTX and BLASTN results with E-values equal or less than 10^-5 ^were treated as 'significant matches', whereas ESTs with no hits or matches with E-values more than 10^-5 ^to proteins in NCBI were classified as 'no significant matches'. The ESTs were mapped to Gene Ontology (GO) by using Blast2GO [21,22] and summarized according to their molecular functions, biological processes and cellular components. Differentially expressed transcripts in multiple oil palm cDNA libraries were detected by using R statistics [6] with Bonferonni correction at the significance threshold of 2.35 × 10^-5 ^using the web tool IDEG6 [23,24].

### Northern analyses

RNA blots were prepared from 10 μg total RNA from shoot apical meristems and flowers of different developmental stages. These blots were hybridised in 0.5 M SSPE pH 7.2, 1 mM EDTA, 7 % (w/v) SDS, 1 % (w/v) BSA and 100 μg/ml denatured herring sperm DNA and ^32^P-dCTP labelled probe at 60°C (for EgSBP) or 65°C (for EgSEP) for overnight. The membranes were washed in 40 mM sodium phosphate buffer, 5%(w/v) SDS for 10 min at 60°C (for EgSBP) or 65 °C (for EgSEP) twice, then followed by washing in 40 mM sodium phosphate buffer, 1 % (w/v) SDS at room temperature for 10 min. Subsequently, the membranes were exposed to autoradiography film for five days.

## Authors' contributions

CLH, HK, SHT, SSRSA and MOA contributed to the conception and design of the study and coordinated the study. YYK, MCC, SST, WHN, KAL, YPL, SEO and JMT were involved in the generation of oil palm ESTs. CLH and WWL analysed the data. CLH drafted and revised the manuscript. All authors have read and approved the final manuscript.

## Supplementary Material

Additional file 1 Sequences of oil palm TUCs. Click here for file
